# A Comparison of the Microbial Production and Combustion Characteristics of Three Alcohol Biofuels: Ethanol, 1-Butanol, and 1-Octanol

**DOI:** 10.3389/fbioe.2015.00112

**Published:** 2015-08-06

**Authors:** Florian Kremer, Lars M. Blank, Patrik R. Jones, M. Kalim Akhtar

**Affiliations:** ^1^Institute for Combustion Engines (VKA), RWTH Aachen University, Aachen, Germany; ^2^Aachen Biology and Biotechnology (ABBt), Institute of Applied Microbiology (iAMB), RWTH Aachen University, Aachen, Germany; ^3^Department of Life Sciences, Imperial College London, London, UK; ^4^Institute of Molecular Plant Sciences, School of Biological Sciences, University of Edinburgh, Edinburgh, UK

**Keywords:** biofuels, alcohols, 1-octanol, 1-butanol, combustion, engine

## Abstract

Over the last decade, microbes have been engineered for the manufacture of a variety of biofuels. Saturated linear-chain alcohols have great potential as transport biofuels. Their hydrocarbon backbones, as well as oxygenated content, confer combustive properties that make it suitable for use in internal combustion engines. Herein, we compared the microbial production and combustion characteristics of ethanol, 1-butanol, and 1-octanol. In terms of productivity and efficiency, current microbial platforms favor the production of ethanol. From a combustion standpoint, the most suitable fuel for spark-ignition engines would be ethanol, while for compression-ignition engines it would be 1-octanol. However, any general conclusions drawn at this stage regarding the most superior biofuel would be premature, as there are still many areas that need to be addressed, such as large-scale purification and pipeline compatibility. So far, the difficulties in developing and optimizing microbial platforms for fuel production, particularly for newer fuel candidates, stem from our poor understanding of the myriad biological factors underpinning them. A great deal of attention therefore needs to be given to the fundamental mechanisms that govern biological processes. Additionally, research needs to be undertaken across a wide range of disciplines to overcome issues of sustainability and commercial viability.

## Introduction

Over the last decade, microbes have been engineered for the manufacture of a variety of biofuels. Both ethanol and 1-butanol have notable histories that stretch back to more than a century and only in modern times have their production, via microbial approaches, been revived (Green, 2011; Buchholz and Collins, 2013). These alcohols have great potential as transport biofuels. Their linear hydrocarbon backbones, as well as oxygenated content, confer combustive properties that are suitable for use in internal combustion engines. Potential fuel candidates can, however, be extended to other saturated alcohols and one relatively new promising candidate is 1-octanol. Its synthesis, which has recently been demonstrated using engineered microbes, offers excellent compatibility with diesel engines (Heuser et al., 2013; Akhtar et al., 2015). Herein, we summarize research findings relating to the microbial synthesis of ethanol, 1-butanol, and 1-octanol; and describe their combustion characteristics in internal combustion engines, with particular emphasis given to 1-octanol.

## Biological Production

Ethanol is the most produced biofuel with a worldwide production of ~25 billion tonnes per year (RFA, 2015). Under oxygen-limiting conditions, ethanol synthesis plays an important role in maintaining the redox balance of the host organism, via the regeneration of NAD^+^. It is this redox role that permits ethanol to be produced with great efficiency (>90%). Commercial manufacturing of ethanol largely involves yeast fermentation of sugar-based feedstocks, resulting in titers of up to 8–12% (v/v) over a 6–10 h fermentation period (Basso et al., 2011). The range of substrate feedstock, however, is constrained by the host metabolism, which can be overcome by employing alternative host organisms. Ingram et al. (1987) first demonstrated this by transferring the ethanol-producing *Zymomonas mobilis* pathway, comprising pyruvate decarboxylase and alcohol dehydrogenase, to *Escherichia coli*. Since this seminal study, ethanol production has been shown for a variety of carbon feedstocks and in a broad range of hosts (Nicola et al., 2011; Gao et al., 2012). Although microbial production of ethanol can be highly productive and efficient, there are concerns in the choice of ethanol as a fuel target on account of its hygroscopic nature and oxygen solubility, which can trigger stress corrosion cracking of steel (Kane and Maldonado, 2004). Due to the poor compatibility of ethanol with the current fuel infrastructure, much of the attention has therefore shifted toward the production of the alcohol fuel, 1-butanol.

Clostridial species serve as the most commercially viable platforms for the renewable generation of 1-butanol (Green, 2011). For a comprehensive review on the 1-butanol pathway, refer to Gheshlaghi et al. (2009). To date, metabolic engineering in conjunction with bioprocess optimization have resulted in high-performance clostridial strains capable of generating as much as 152 g L^−1^ of 1-butanol (Köhler et al., 2015). As with ethanol synthesis, the efficiency of butanol production is also very high since it helps to maintain the redox balance of the host metabolism (~90%). Refined approaches have led to the utilization of a variety of diverse carbon feedstocks and, in some cases, diversion of the by-products to commercially relevant products, such as isopropanol (Lee et al., 2011; Ellis et al., 2012; Raganati et al., 2013; Jiang et al., 2014). A major drawback with clostridal cells is their loss of viability upon exposure to ambient air, which makes genetic manipulations with this organism a cumbersome and finicky task (Zhu et al., 2011). Furthermore, pathway optimization is limited by the range of genetic tools and protein expression systems. In recognizing these constraints, Inui et al. (2008) and Atsumi et al. (2008) transferred the clostridial genes encoding for the 1-butanol pathway to the more genetically amenable host, *E. coli*, and demonstrated that 1-butanol synthesis was achievable. Later, breakthroughs led to high-performance pathways resulting in titers of up to 30 g L^−1^ (Bond-Watts et al., 2011; Shen et al., 2011). To further extend the range of substrate feedstock, a number of research groups have transferred the 1-butanol pathway to alternative host organisms, such as yeast, cyanobacteria, and methylobacteria, all with distinct metabolic features (Steen et al., 2008; Lan and Liao, 2011; Hu and Lidstrom, 2014) and even employed alternative metabolic routes to improve oxygen tolerance (Pásztor et al., 2015).

In contrast to 1-butanol, 1-octanol is not known to accumulate in substantial amounts in any known organism and is found in trace amounts as a volatile metabolite in certain microbes (Elgaali et al., 2002; Hamilton-Kemp et al., 2005). Detailed insights into volatile metabolites have only recently been gained with the study of yeast (Halbfeld et al., 2014). Of the 93 metabolites reported in the literature, only 19 have entries in comprehensive biochemical databases. Interestingly, these metabolites [refer to Table 1 of Halbfeld et al. (2014) for a comprehensive list] could provide new direction with respect to the production of fuels. Further investigation will, however, be required of their physicochemical and fuel characteristics to gage their suitability for commercial applications. Nonetheless, a handful of studies, all within the past 3 years, have already demonstrated the synthesis of 1-octanol in microbes. By extending the 1-butanol pathway, James Liao’s research team was able to show that the synthesis of higher chain alcohols was biologically feasible and reported ~70 mg L^−1^ of 1-octanol (Machado et al., 2012). In another study, based on the process of reverse β-oxidation reported by Inui et al. (1984) in which carbon chain elongation proceeds via acyl-CoA intermediates, Dellomonaco et al. (2011) obtained ~100 mg 1-octanol L^−1^. By applying this principle of fatty-acid chain elongation instead with fatty acyl ACP intermediates, Akhtar et al. (2015) reported a titer of ~62 mg L^−1^, which was developed without any attempt at chromosomal engineering hinting at the possibility that extensive genetic modifications could lead to higher titers. Since this pathway depends on the host’s essential fatty-acid machinery, a fine balance between host viability and synthesis of the fatty-acid derived 1-octanol would need to be established to ensure high productivity. This same study also demonstrated that 1-octanol could be exclusively excreted into the culture media and that AcrAB-TolC efflux pump may be responsible for the excretion. In addition to fatty-acid metabolism, alcohols (comprising both linear and branched groups) can also be derived from pathways linked to amino acid metabolism, in this case threonine, as demonstrated by Zhang et al. (2008). By inducing up to 11 recombinant enzymes in a threonine-producing host, a titer of ~2 mg L^−1^ 1-octanol was obtained (Marcheschi et al., 2012). Interestingly, with octane as the carbon source, 1-octanol productivity of 1.3 g day^−1^ was found to be possible using an active biofilm consisting of a Pseudomonas strain harbouring a recombinant P450 monooxygenase (Gross et al., 2012). Such an approach, however, would only be relevant for chemical manufacturing purposes, rather than development of a renewable platform for biofuel production.

**Table 1:** **Definition of key terminologies**.

Terminology	Definition
Autoignition	The spontaneous ignition of a fuel-air mixture which occurs when initially slow thermal reactions have a large enough chain-branching component to sustain and accelerate oxidation. The increasing concentration of radicals along with the reaction rate eventually lead to a rapid explosive rise in radical concentration, oxidation rate, and temperature – ignition!
Autoignition point	The lowest temperature at which self-ignition of an air-fuel mixture occurs (without the aid of an external ignition source)
Cetane number (CN)	This refers to the reactivity/autoignition behavior of fuels for diesel/CI-type combustion and is described in norm EN 590. This value is in relation to cetane (CN = 100) and alpha-methyl naphthalene (CN = 0). Thus, a CN of 52 means that the tested fuel has the same autoignition property as a volumetric mixture of 52% Cetane and 48% alpha-methyl naphthalene. The higher the CN, the higher the autoignition tendency
Heat of vaporization	The amount of (heat) energy to completely vaporize 1 mole/kg of fuel
Kinematic viscosity	The viscosity of fluid describes its resistance toward shear stress. These values are typically given either as “dynamic” or “kinematic” viscosity; the difference between both is just the density of the fluid
Knocking combustion	The undesired self-ignition in the unburned air/fuel mixture, which has not yet been reached by the flame front, leads to high-pressure fluctuations and possibly severe engine damages
Kow	Also known as the partition water coefficient. This is the ratio between the concentration of a chemical in *n*-octanol and water at equilibrium at a specified temperature (i.e., concentration of chemical in octanol/concentration of chemical in water)
Lubricity	The fuel’s ability to lubricate two moving material partners. Especially for CI fuels (e.g.*diesel*), the lubricity of a fuel needs to be taken into account as the high-pressure fuel pump is lubricated by the fuel itself and not the engine-oil
Metabolic yield efficiency	This is the ratio between the observed and the theoretical yield (i.e., observed yield of pathway/theoretical yield of pathway*100)
Research octane number (RON)	This refers to the knocking resistance of fuels for gasoline/SI-type combustion and is described in norm EN 228. This value is in relation to iso-octane (RON = 100) and *n*-heptane (RON = 0). Thus, a RON of 95 means that the tested fuel has the same anti-knocking properties as a volumetric mixture of 95% iso-Octane and 5% *n*-heptane. The higher the RON, the higher the knocking resistance
Vapor pressure	This is the pressure, within a closed system and at a given temperature, exerted by a vapor in thermodynamic equilibrium with its solid or liquid state phase

To date, there have been far fewer studies on 1-octanol production in comparison to both ethanol and 1-butanol. The biological production of 1-octanol is clearly inefficient, and there are several possible reasons for this. First, native pathways of the host organism may divert the desired intermediates away from 1-octanol. There is a greater likelihood of this for 1-octanol synthesis, since it involves repeated cycles of reactions (to form the eight-carbon backbone), whereas for ethanol/1-butanol only a single cycle of reactions is required (to form the two- and four-carbon backbones, respectively). The use of mutant hosts to eliminate diverting/competing pathways for 1-octanol production has yet to be identified. Second, poor kinetics/substrate specificities of the enzymes involved may lead to pathway bottlenecks. The CAR enzyme, for example, exhibits poor catalytic efficiency with short-chain substrates (octanoic acid; Kcat/Km; ~0.5) compared to long-chain substrates (dodecanoic acid; Kcat/Km; ~2) (Akhtar et al., 2013). In such cases, protein engineering may provide an effective solution to alleviate these bottlenecks. Finally, the physicochemical characteristics of 1-octanol and its precursor metabolites may limit reaction rates and/or even excretion into the media. This could be overcome using well-established biological strategies. A good example is the spatial arrangement of enzymes, via DNA/RNA aptamers or protein scaffolds, for localizing the metabolites within the vicinity of the enzyme active sites (Lee et al., 2012).

## Combustion Characteristics

Ethanol and 1-butanol have long been known as alternative engine fuels, as their high heating values (19.6 MJ L^−1^ for ethanol; 29.2 L^−1^ for 1-butanol) allow applicability for existing gasoline-dependent, spark iginition (SI) engines (Thewes et al., 2012; Sarathy et al., 2014). Their compact molecular structures result in high research octane numbers; ethanol (RON 106) and 1-butanol (RON 94 to 96); which are close to or exceed that of standard gasoline (RON 95); descriptions of key terminologies are provided in Table 1. Higher octane values improve engine performance by reducing the abnormal combustion phenomenon, known as engine “knocking”. Another important fuel parameter particularly for SI engines is vapor pressure since low or high vapor pressures can complicate engine start-ups or even lead to engine stalls. Both ethanol and 1-butanol are disadvantageous in this regard since their exceedingly low vapor pressures impose combustion limitations under cold conditions (<0°C). To overcome this, SI engines designed to run on alcohols require gasoline supplementation. However, the higher latent heat of vaporization for both alcohols, which exceeds that of gasoline by a factor of up to twofold, can lead to an internal cooling of the combustion mixture. This decreases the knocking tendency of the fuel, especially within modern, direct-injection SI engines. The overall combination of these effects can result in an increase in the combustion efficiency of SI engines compared with standard gasoline, though the mixture formation of fuel and air can be worsened, which can lead to higher hydrocarbon and particulate emissions (Thewes et al., 2012).

A clear advantage in the use of 1-octanol as a fuel compared to ethanol and 1-butanol is its higher energy density (33.7 MJ L^−1^). Experimental trials have so far shown 1-octanol as a promising fuel candidate with diesel-like properties that could be applied to compression-ignition (CI) engines in an unblended state (Heuser et al., 2013). Within a CI engine, the combustion process is initiated by self-ignition of the air and fuel mixture, rather than the spark-assisted combustion of gasoline engines. This process, which depends on the temperature and pressure of the in-cylinder gas as well as the degree of chemical reactivity of the fuel, is typically referred to as self-ignition tendency and is quantified by the cetane number, where a high cetane number indicates a high self-ignition tendency. Although 1-octanol has a lower cetane rating of 34 compared to diesel fuel (~54; EN590 standard), it is enough for self-ignition in the CI engine once the engine has warmed up. However, under cold-temperature conditions, an external heat source, such as a glow-plug, may be required on account of the low vapor pressure of 1-octanol. In addition, the lower self-ignition tendency has an indirect beneficial effect on mixture formations. As the lower reactivity leads to a longer time between the start of the injection of the fuel into the engine cylinder and the actual ignition/combustion event, more time is available for the fuel and air to mix throughout the cylinder, which overall leads to better homogenization of the fuel/air mix. This again, at constant nitrogen oxide emissions, is responsible for a decrease in soot emissions as these are exclusively formed in cylinder areas with an excess of fuel in comparison to the available local air. On the other hand, as the overall nitrogen oxide/soot trade-off is improved, the engine may also be calibrated in a way that a reduction of nitrogen oxide and soot can be realized at the same time.

The self-ignition tendency, the viscosity, and boiling behavior all have a major impact on the combustion characteristics of diesel-type fuels as they directly influence the mixture formation between fuel and air. The fuel viscosity is particularly important as it dictates the spray pattern and atomization of the injected fuel. In general, a low viscosity and low boiling curve lead to a beneficial effect especially on the soot particle emissions of the CI engine (Janssen et al., 2011). The fuel viscosity of 1-octanol (kinematic viscosity; 4.4 mm^2^ s^−1^) is close to the upper limit of the diesel viscosity range (1.8–5.8 mm^2^ s^−1^), while the boiling point of 1-octanol (195°C) is at the low end of the diesel boiling curve (180–300°C); the latter property allows the fuel to evaporate faster (Janssen et al., 2011). Overall, the combined effect of the low boiling point and the reduced cetane number overcompensates the negative influence of the higher kinematic viscosity of 1-octanol and ensures good fuel/air mixture formation for combustion purposes.

## Concluding Remarks

The benchmarks for the biological production of ethanol, 1-butanol, and 1-octanol as well as their physicochemical and fuel characteristics are summarized in Table 2. All three alcohol fuels enhance engine performance. Of the three fuels considered in this article, we would conclude that for SI engines, ethanol is the most suitable choice, while for CI engines it would be 1-octanol. However, it is worth pointing out that the use of ethanol is drastically offset by its high affinity for water, which can promote steel corrosion cracking and, in this regard, 1-butanol may offer a better prospect with regard to pipeline compatibility (Kane and Maldonado, 2004). Currently, the metabolic engineering of organisms for 1-octanol lags some way behind that of ethanol and 1-butanol. It is quite likely that as our understanding of biological systems increases in tandem with developments in engineering tools, the differences in production performances between these systems will become smaller over time. Already, within 2 years, there has been a substantial improvement in the titers of longer-chain (C_12_–C_16_) alcohols from ~360 mg L^−1^ to ~1.6 g L^−1^ (Akhtar et al., 2013; Youngquist et al., 2013). Thus, if progress in the microbial synthesis of 1-octanol parallels that of longer-chain alcohols, then we can expect significant developments in the very near future.

**Table 2:** **Current benchmarks for the biological production of ethanol, 1-butanol, and 1-octanol along with their physicochemical and fuel characteristics**.

	Fossil fuel		Biofuel		
	Petroleum	Petrodiesel	Ethanol	1-Butanol	1-Octanol
Examples of engineered host organisms	n/a	n/a	*Saccharomyces cerevisiae, Zymomonas mobilis, E. coli, Klebsiella oxytoca, Pseudomonas putida, Thermoanaerobacter, Fusarium oxysporium, Cyanobacteria, Microalgae*	*Clostridum, E. coli, Saccharomyces cervesiae, Pseudomonas putida, Bacillus subtilis, Lactobacillus brevis, Pyrococcus furious, Methylobacterium extorquens, Cyanobacteria*	*E. coli*
Naturally evolved pathway	n/a	n/a	Yes	Yes	No
Research and development (years)	n/a	n/a	~100 years	~100 years	~3 years
Achieved titers (g L^−1^)	n/a	n/a	100–228^a^	20^b^	0.1^c^
Achieved productivity (g L^−1^ h^−1^)	n/a	n/a	10–23^a^	0.27^b^	0.004^c^
Cultivation method	n/a	n/a	Bioreactor^a^	Bioreactor^b^	Shake-flask^c^
Metabolic yield efficiency* (%)	n/a	n/a	>90	90	7
Energy content (MJ L^−1^)	32.1	40.3	19.6	29.2	33.7
Water solubility (g L^−1^)^d^	Immiscible	Immiscible	Miscible	79	0.46
Kow^d^	~4.1–6.8	~5.3–8	−0.03	0.8	3.1
Boiling point (°C)^d^	~27–225	~150–350	78	117	195
Freezing point (°C)^d^	~−60	~−12	−114	−90	−16
Heat of vaporization (kJ kg^−1^)^d^	425	~300–330	912	702	545
Autoignition (°C)^e^	246–280	177–329	420	343	270
Density (g cm^−3^)^a^	~0.82 (avg)	~0.84 (avg)	0.79	0.81	0.83
Engine application	SI	CI	SI	SI	CI
Research octane number^f^	95	n/a	106	96	<70
Cetane number^e^	n/a	54	11	17	39
Lubricity^g^ (μm corrected wear scar diameter in HFRR test)	711–1064	315	603	623	404
Vapor pressure (mmHg)	275–475	<0.4	55	7	0.08
Kinematic viscosity^h^ (mm^2^ s^−1^)	0.4–0.8@20°C	1.8–5.8@40°C	1.1@40°C	1.7@40°C	4.4@40°C

Values obtained from the following references:

^a^Nicola et al. (2011),

^b^Köhler et al. (2015),

^c^Akhtar et al. (2015), without *in situ* product removal. Precise values will vary depending on the cultivation methods and feedstocks employed. Refer to references ^a–c^for optimum values. Physicochemical and combustion characteristics obtained from the following references:

^d^Haynes (2012),

^e^Harnisch et al. (2013),

^f^Wallner et al. (2013),

^g^Weinebeck and Murrenhoff (2013), and

*^h^Viswanath et al. (2007). Physicochemical and fuel characteristics of petroleum and petrodiesel are included for comparison*.

**Relative to theoretical yield.

*K_OW_, octanol/water partition coefficient; SI, spark-ignition; CI, compression-ignition*.

Aside from metabolic modifications, extrinsic factors, such as biological toxicity, will also influence biofuel productivity. While microbes are able to withstand ~15% (v/v) ethanol, maximal tolerance toward 1-butanol usually lies around 2–3% (v/v) (Knoshaug and Zhang, 2009; Kanno et al., 2013). In contrast, bacteria, such as *P. putida*, harbor cellular mechanisms, which permit growth in the presence of a secondary phase of 1-octanol, suggesting that a higher tolerance level is likely to be achievable for systems engineered for 1-octanol production (Rojas et al., 2004; Blank et al., 2008; Segura et al., 2012). *In situ* product removal techniques have also been explored in great depth for biological systems that produce ethanol- and 1-butanol, though not 1-octanol (Onuki et al., 2008; Dhamole et al., 2012; Xue et al., 2014). It could be speculated that the low solubility of 1-octanol (460 mg L^−1^) may ease purification from aqueous-based cultivation systems and, in turn, avoid an energy-intensive distillation process typically required for the purification of ethanol and 1-butanol; this is an area that remains to be investigated.

Biofuel candidates are not just limited to linear-chain alcohols but can also be extended to branched-chain alcohols (Zhang et al., 2008). Additionally, other types of biofuels, such as terpenes and alkanes, could also serve as potentially good candidates (Wang et al., 2013; Zhang et al., 2014). Further research will help to ascertain their suitability as transportation fuels. Thus, any conclusions drawn at this stage regarding the most superior biofuel would be premature as there are still many areas that need to be addressed. For example, to improve sustainability, we need to assess the functionality of engineered pathways that have traditionally been engineered in genetically tractable organisms, such as *E. coli*, within photobiological systems. For downstream processing, consideration also needs to be given to the amenability of biofuels to large-scale extraction and purification from aqueous-based cultivation media. Further downstream to this and just as important is a much-needed evaluation on the compatibility of the next generation of drop-in biofuels with combustion engines and the pipeline infrastructure. Also, fuel parameters relating to stability, storage, and handling would need to be taken into account. Currently, there is very little or no information pertaining to these areas for the new generation of drop-in biofuels.

Given the enormous potential of biofuels to reduce the environmental footprint, the authors envision a biofuel economy based on microbial platforms capable of producing infrastructure-compatible fuels, from industrial waste streams or via the fixation of carbon dioxide in the presence of sunlight and water, within controlled and strictly regulated environmental settings. Such an approach would not only ameliorate public attitudes over the use of genetically modified organisms for societal benefit but also eliminate ethical dilemmas concerning the use of agricultural resources for fuel production. The commercialization of biofuels is beset with many challenges, one of which is its economic competitiveness with fossil fuels. The high costs of the feedstock coupled with the low efficiencies/yields/productivities of the current generation of platforms make biofuels prohibitively expensive. This is made more apparent with the recent discovery of shale reserves, which have led to drops in oil prices and forced cutbacks in bioethanol production (World Bank Group, 2015). Even so, continued dependence on fossil fuels is not a viable solution to growing global concerns over the environmental footprint and could lead to detrimental economic consequences in the long-term, particularly with regard to human health and climate change (Watts et al., 2015).

The major challenges in the development and optimization of biological platforms for fuel production stem from our poor understanding of the myriad factors underpinning them. A great deal of attention therefore needs to be given to the fundamental mechanisms that govern biological processes and not just to the translatability of these platforms for commercial purposes. Considering that the biofuel of choice would need to meet strictly defined criteria to overcome issues of sustainability and commercial viability, research needs to be undertaken across a wide range of disciplines. Thus, it is imperative that viewpoints and opinions are actively sought and shared among experts covering different aspects of biofuel research.

## Conflict of Interest Statement

The authors declare that the research was conducted in the absence of any commercial or financial relationships that could be construed as a potential conflict of interest.
